# Assessing self-management in patients with diabetes mellitus type 2 in Germany: validation of a German version of the Summary of Diabetes Self-Care Activities measure (SDSCA-G)

**DOI:** 10.1186/s12955-014-0185-1

**Published:** 2014-12-18

**Authors:** Martina Kamradt, Kayvan Bozorgmehr, Johannes Krisam, Tobias Freund, Marion Kiel, Markus Qreini, Elisabeth Flum, Sarah Berger, Werner Besier, Joachim Szecsenyi, Dominik Ose

**Affiliations:** Department of General Practice and Health Services Research, University Hospital Heidelberg, Voßstr. 2, Geb. 37, D-69115 Heidelberg, Germany; Department of Medical Biometry; Institute of Medical Biometry and Informatics, University Hospital Heidelberg, Im Neuenheimer Feld 305, D-69120 Heidelberg, Germany; Genossenschaft Gesundheitsprojekt Mannheim e.G., Liebfrauenstr. 21, D-68259 Mannheim, Germany

**Keywords:** Diabetes mellitus type 2, Self-management, Self-care, Outcome measurement, German, Validation

## Abstract

**Background:**

One of the most widely used self-reporting tools assessing diabetes self-management in English is the Summary of Diabetes Self-Care Activities (SDSCA) measure. To date there is no psychometric validated instrument in German to assess self-management in patients with diabetes mellitus. Therefore, this study aimed to translate the SDSCA into German and examine its psychometric properties.

**Methods:**

The English version of the SDSCA was translated into German following the guidelines for cultural adaptation. The German version of the SDSCA (SDSCA-G) was administered to a random sample of 315 patients with diabetes mellitus type 2. Reliability was analyzed using Cronbach’s alpha coefficient and item characteristics were assessed. Exploratory and confirmatory factor analysis (EFA and CFA) were carried out to explore the construct validity. A multivariable linear regression model was used to identify the influence of predictor variables on the SDSCA-G sum score.

**Results:**

The Cronbach’s alpha for the SDSCA-G (all items) was α = 0.618 and an acceptable correlation between the SDSCA-G and Self-management Diabetes Mellitus-Questionnaire (SDQ) (ρ = 0.664) was identified. The EFA suggested a four factor construct as did the postulated model. The CFA showed the goodness of fit of the SDSCA-G. However, item 4 was found to be problematic regarding the analysis of psychometric properties. The omission of item 4 yielded an increase in Cronbach’s alpha (α = 0.631) and improvements of the factor structure and model fit. No statistically significant influences of predictor variables on the SDSCA-G sum score were observed.

**Conclusion:**

The revised German version of the SDSCA (SDSCA-G) is a reliable and valid tool assessing self-management in adults with type 2 diabetes in Germany.

**Electronic supplementary material:**

The online version of this article (doi:10.1186/s12955-014-0185-1) contains supplementary material, which is available to authorized users.

## Background

As one of the major non-communicable diseases [1], diabetes mellitus, has become a challenging health problem worldwide [2]. According to estimates of the International Diabetes Federation [2], 382 million adults suffered from diabetes in 2013 (worldwide prevalence: 8.3%). Additionally, there is an increasing prevalence of multiple, co-occurring conditions especially for patients with severe diseases like diabetes mellitus [3,4]. This increasing prevalence influences the delivery of chronic illness care strongly [5,6].

As a chronic disease, diabetes mellitus requires a high level of individual responsibility because the vast majority of daily care is handled by the patient himself [7,8]. Therefore, care for people with chronic diseases is shifting away from patients as passive recipients to patients with active involvement in their everyday health care. To accomplish this change strengthening self-management has become a major task in the health care sector [8-11], which is necessary to meet the needs of chronically ill people and achieve better health care outcomes [9-11].

Self-management is a multidimensional construct and in general defined as „[…] the individual’s ability to manage the symptoms, treatment, physical and psychosocial consequences and life style changes inherent in living with a chronic condition“ [11]. Self-management is embedded in the broad concept of self-care which refers to the ability to care for oneself and perform activities necessary to achieve, maintain or promote optimal health [12]. In the literature, self-management has been conceptualized as a subset of self-care [11,13] but also self-care is frequently subsumed under the concept of self-management [14]. Concepts like self-efficiency or empowerment are strongly related with self-management and self-care because attributes related with these concepts influence patients’ behavior as well as enables patients to be actively involved in their daily care [9,12,15].

Particularly in diabetes care, self-management skills are necessary to enable patients managing their own disease. Nutritional management, exercise and physical activity, blood glucose monitoring as well as medication utilization are some major aspects regarding self-management in diabetes [16]. So far, effective self-care behavior has been shown to improve several outcomes of patients with diabetes, e.g. glycemic control, diabetes complications, quality of life and dietary habits [7,10,17,18].

The important question with regard to self-management in diabetes care is not whether, but how, these people manage their daily life with their individual health challenges [7]. Therefore, a valid and reliable tool, which assesses self-management behavior in patients with diabetes, is needed. The Summary of Diabetes Self-Care Activities measure (SDSCA) from Toobert and colleagues [8] is one of the most popular and frequently used tools in English-speaking regions. The questionnaire is an 11 item self-reporting tool assessing levels of self-care in adults with diabetes. Several studies have evaluated the SDSCA and shown satisfactory psychometric properties [8,19].

To date, there is no psychometric validated instrument to assess self-management in patients with diabetes mellitus in Germany. Regarding the importance of self-management, it is essential to evaluate this aspect of diabetes care, especially when assessing the effectiveness of specific health care strategies for chronic diseases like diabetes mellitus. In fact, Petrak and colleagues [20] used previously a German version of the SDSCA in their study, but they did not report on the translation process or on the exploration of psychometric properties. Additionally, Schmitt and colleagues [19] designed a questionnaire to assess four well-defined specific self-care-activities associated with glycemic control in Germany. Only items of self-care activities which show relevant association with glycemic control were covered by this instrument. For that reason, several self-care activities which may be of interest in regard to diabetes care were not included. The published preliminary evidence for this instrument by Schmitt and colleagues [19] showed that the study sample was not representative of the general diabetic population. Accordingly, the results of the first psychometric evaluation of this instrument are currently not generalizable.

Hence, the aim of this study was to translate the SDSCA into German and examine its psychometric properties in order to provide an adequate tool and facilitate the collection of appropriate data.

## Methods

### Participants

Participants in this cross-sectional study were randomly recruited from the overall pool of patients with diabetes type 2 in 20 primary care practices (PCPs) located in Germany. The participating PCPs received a list with the inclusion and exclusion criteria for patients along with a screening list with random numbers. Based on the inclusion and exclusion criteria PCPs were asked to create a list of all potentially eligible patients registered in their practice software. In a next step, PCPs selected patients from this list according to the sequences indicated by random numbers. The randomly selected patients were contacted and asked to participate in the study. The procedure was repeated until at least 15 patients per PCP were recruited.

The final study sample consisted of 315 patients who met the following inclusion criteria: diabetes mellitus type 2 (ICD 10: E11-E14) and at least two additional chronic diseases. These inclusion criteria ensured that the study sample consisted of diabetes patients who would benefit most from a high level of self-management and strongly need to be considered when assessing the effectiveness of health care strategies for chronic conditions like diabetes. Therefore, eligible participants had to have at least two co-occurring chronic diseases in addition to diabetes mellitus type 2. Patients, who fulfilled the following criteria were excluded: younger than 18 years, emergency cases as well as suffering from severe acute psychiatric disorders, mental and behavioral disorders due to psychoactive substance use except for alcohol and tobacco use, dementia, malignant neoplasm undergoing current chemotherapy or radiotherapy, transplanted organ/tissue status, care involving dialysis, insurmountable language and communication problems.

The study was approved by the ethics committee of the Medical Faculty of the University of Heidelberg, Germany (application number: S-297/2013) and informed consent was obtained from all patients.

### Instruments

Recruited patients were asked to complete a series of questionnaires after giving consent to participate. The questionnaires included a German version of the SDSCA (SDSCA-G), Self-management Diabetes mellitus-Questionnaire (SDQ) [21], as well as questions regarding socio-demographic aspects and medical treatment (e.g. enrollment in a disease management program (DMP), insulin treatment).

### Summary of Diabetes Self-Care Activities measure (SDSCA)

The SDSCA is a questionnaire which assesses levels of self-care in adults with diabetes and was developed by Toobert and colleagues [8] in the U.S.. The tool contains 11 items, which measure the frequency of performing diabetes self-care activities over the last seven days including diet, exercise, blood glucose testing, foot care and tobacco use. The respondent marks the number of days on which the indicated behavior was performed on an eight-point Likert scale to answer the questions. The first ten items are summed to a total score as well as to four sub scores: diet (item 1–4), exercise (item 5–6), blood-glucose testing (item 7–8) and foot-care (item 9–10). The eleventh item focuses on smoking habits and assesses the average number of cigarettes smoked per day [8,19].

### Self-Management Diabetes Mellitus-Questionnaire (SDQ)

The Self-Management Diabetes Mellitus-Questionnaire is a brief tool with four items assessing self-care activities regarding diet, blood glucose monitoring and foot care. Each item is scored on a five-point Likert scale indicating the frequency of the specified behavior as 0 = never to 4 = always. This questionnaire was adapted and used in a recent cluster randomized controlled trial by Freund and colleagues [21]. The original tool was developed by Peeters and colleagues [22].

### Translation procedure

The translation process followed the guidelines for cultural adaption in order to assure content validity [23,24]. A description on how the content validity was established will be given in the following subsection “statistical analyses”. The forward translation of the SDSCA into German was done by two researchers (TF and EF), who were aware of the objectives of the SDSCA. Each researcher translated the questionnaire independently. Backwards translation (from German to English) was carried out by a native English speaker (SB), who had no prior knowledge of the instrument. The translations were compared and differences discussed in the translation team to reach consensus. During the whole translation process, the overall aim was to ensure comprehensibility and capture the original idea of each item instead of a rigid literal translation without adaption the cultural concept (see Table 1).

**Table 1:** **The Summary of Diabetes Self-care Activities Measure – Original and translated German version**

	**Items**	
	**Original [8]**	**German**
	*Diet*	*Ernährung*
1	How many of the last SEVEN DAYS have you followed a healthful eating plan?	An wie vielen von den letzten SIEBEN TAGEN haben Sie sich gesund ernährt?
2	On average, over the past month, how many DAYS PER WEEK have you followed your eating plan?	An wie vielen TAGEN PRO WOCHE im letzten Monat haben Sie sich im Durchschnitt gesund ernährt?
3	On how many of the last SEVEN DAYS did you eat five or more servings of fruit and vegetables?	An wie vielen der letzten SIEBEN TAGE haben Sie 5 oder mehr Portionen Obst oder Gemüse gegessen?
4	On how many of the last SEVEN DAYS did you eat high fat foods such as red meat or full-fat dairy products?	An wie vielen der letzten SIEBEN TAGE haben Sie fetthaltige Produkte wie rotes Fleisch oder nicht fettreduzierte Milchprodukte gegessen?
	*Exercise*	*Körperliche Aktivität*
5	On how many of the last SEVEN DAYS did you participate in at least 30 minutes of physical activity? (Total minutes of continuous activity, including walking)	An wie vielen der letzten SIEBEN TAGE haben Sie sich mindestens 30 Minuten lang am Stück körperlich betätigt (auch Spazierengehen, Garten- oder Hausarbeit)?
6	On how many of the last SEVEN DAYS did you participate in a specific exercise session (such as walking, biking) other than what you do around the house or as part of your work?	An wie vielen der letzten SIEBEN TAGE haben Sie Sport getrieben (z.B. Schwimmen, Nordic Walking, Radfahren)?
	*Blood Sugar Testing*	*Blutzuckertest*
7	On how many of the last SEVEN DAYS did you test your blood sugar?	An wie vielen der letzten SIEBEN TAGE haben Sie Ihren Blutzucker gemessen?
8	On how many of the last SEVEN DAYS did you test your blood sugar the number of times recommended by your health care provider?	An wie vielen der letzten SIEBEN TAGE haben Sie Ihren Blutzucker so oft gemessen, wie man es Ihnen von medizinischer Seite empfohlen hat?
	*Foot Care*	*Fußpflege*
9	On how many of the last SEVEN DAYS did you check your feet?	An wie vielen der letzten SIEBEN TAGE haben Sie Ihre Füße untersucht?
10	On how many of the last SEVEN DAYS did you inspect the inside of your shoes?	An wie vielen der letzten SIEBEN TAGE haben Sie die Innenseite Ihrer Schuhe kontrolliert?
	*Smoking*	*Rauchen*
11	Have you smoked a cigarette – even one puff – during the past SEVEN DAYS?	Haben Sie in den letzten SIEBEN TAGEN eine Zigarette – auch nur einen Zug – geraucht?

### Statistical analyses

The statistical analyses were carried out using IBM SPSS 21 [25].

Item characteristics were analyzed by calculating item difficulty indices (defined as percentage of missings per item), inter-item-correlations, item-subscale-correlations and item-total correlation. Every item was analyzed using the scale’s reliability coefficient (Cronbach’s α) in case the item was deleted.

In order to evaluate the scale’s structure, exploratory principal component factor analysis (EFA) by employing the varimax rotation method was performed.

Confirmatory factor analysis (CFA) was performed using AMOS 22.0 [26] to assess the model fit defined by the four factors diet (items 1–4), exercise (items 5 and 6), blood glucose testing (items 7 and 8) and foot care (items 9 and 10) using the maximum likelihood method.

The convergent content validity of the SDSCA-G was assessed based on the correlation (Spearman’s rho) of its sum score with the sum score of the SDQ [21], which has an acceptable internal consistency .

Possible differences between migrants and non-migrants regarding self-care behavior were assessed by comparing the mean SDSCA-G of the two-groups by applying a two-sample t-test. The migration status of a patient was determined by the tool provided by Schenk and colleagues [27].

A multivariable linear regression model was used with the aim to identify the influence of predictor variables on the SDSCA-G sum score. The model included sex, DMP enrollment (yes/no) and migration status as binary factors while age and number of comorbidities were included as continuous covariates.

Due to the exploratory character of the study, all resulting p-values were to be interpreted in a descriptive manner. A p-value smaller than 0.05 was regarded as statistically significant.

## Results

A total of 315 patients were included in the study. The mean age of the population was 71.6 ± 9.2 years, 41.2% of the patients were female, 33.6% were treated with insulin and the mean number of comorbidities was 2.8 ± 1.6. 36.2% of the patients were enrolled in a DMP and 14.3% had a migration background (see also Table 2).

**Table 2:** **Patient characteristics**

**Variable**	
**Gender (N = 306)**	
Male	180 (58.8%)
Female	126 (41.2%)
**Age in years (N = 308)**	
Mean ± SD, range	71.58 ± 9.17, 44.0-91.0
**Treatment with insulin (N = 298)**	
No	198 (66.4%)
Yes	100 (33.6%)
**DMP participation (N = 268)**	
No	97 (36.2%)
Yes	171 (63.8%)
**Additional chronic diseases** ^**a**^ **(N = 315)**	
Hypertension	233 (74.0%)
Coronary heart disease	149 (47.3%)
Arthrosis/rheumatoid arthritis	137 (43.5%)
Chronic back pain	108 (34.3%)
Asthma/chronic obstructive pulmonary disease	54 (17.1%)
Chronic renal disease	40 (12.6%)
Depression	39 (12.3%)
Others	25 (7.9%)
Cancer	24 (7.6%)
Chronic dermatitis/allergy	22 (7.0%)
Chronic gastrointestinal disease	20 (6.3%)
Anxiety disorder	12 (3.8%)
Parkinson’s disease	7 (2.2%)
**Migration background** ^**b**^ **(N = 308)**	
No	264 (85.7%)
Yes, first generation migrant	38 (12.3%)
Yes, second generation migrant	6 (1.9%)
Yes, third generation migrant	0 (0.0%)

SD: standard deviation.

^a^Additional chronic diseases are patient reported.

^b^Migration status and migration generation was assessed based on algorithm provided in [27].

### Assessment of item characteristics and reliability

The mean score of the items 1–10 of the SDSCA-G was 3.48 with a standard deviation of 1.38, while the mean item difficulty was 0.0702 ± 0.0271. Our analysis revealed a mean item-subscale correlation of 0.481 ± 0.250 and a mean item-total correlation of 0.292 ± 0.141. With the exception of items 4 and 5, the deletion of any other item yielded a decrease in the sum scale’s alpha coefficient (see Table 3), which had an acceptable value of α = 0.618 and is comparable to the reliability coefficient of α = 0.63 reported by Schmitt and colleagues [19]. The internal consistency of the blood glucose testing subscale was excellent (α = 0.947), while the foot care subscale’s consistency was acceptable (α = 0.607). For the subscales diet and exercise, a relatively poor alpha coefficient was observed (α = 0.566 and 0.498, respectively). A detailed overview of the item characteristics is depicted in Table 3. These results show that the discrimination of the items is acceptable, with the exception of item 4 which showed a relatively low item-subscale and item-total correlation. The relatively large standard deviation of the items associated with blood glucose testing stems supposedly from the fact that both insulin-dependent and insulin-independent patients (see Table 2) were enrolled in the study. The item 11 which determines the smoking status was not incorporated into the sum score. It had an item difficulty of 0.003 and revealed that 8.6% of the study population were smokers.

**Table 3:** **Distribution of item scores, item difficulty, item-subscale and item-total correlation and internal consistency in case of deletion**

**Item**	**Distribution of item scores** ^**a**^	**Item difficulty** ^**b**^	**Item-subscale-correlation** ^**c**^	**Item-total-correlation** ^**c**^	**α if item deleted**
1	4.91 ± 1.904	0.0952	0.510	0.446	0.565
2	4.90 ± 1.786	0.1016	0.532	0.397	0.576
3	4.25 ± 2.252	0.0667	0.310	0.251	0.601
4	4.24 ± 2.066	0.0603	0.112	0.088	0.631
5	4.40 ± 2.373	0.0476	0.336	0.067	0.640
6	1.41 ± 1.987	0.0603	0.336	0.161	0.617
7	2.92 ± 3.055	0.0508	0.899	0.400	0.562
8	2.70 ± 3.029	0.1238	0.899	0.394	0.564
9	3.54 ± 2.725	0.0444	0.440	0.386	0.567
10	1.51 ± 2.306	0.0508	0.440	0.329	0.583

^a^mean ± standard deviation, ^b^defined as percentage of missings, ^c^Pearson coefficient, part-whole-corrected.

### Exploratory factor analysis

The exploratory factor analysis including N = 237 patients suggested a four factor model, supported by the scree test, which explained 68.1% of the variance. The varimax rotation converged within 6 iterations. With the exception of item 4, which formed a factor together with the two items 5 and 6 measuring exercise, EFA yielded the postulated model. The rotated component matrix displaying factor loadings is given in Table 4. It should be noted that the primary analysis population of N = 237 consists only of those patients who had no missing values in all of the first 10 items of the SDSCA-G. In order to assess the robustness of the achieved results, another exploratory factor analysis was conducted on the full set of N = 315 patients as a sensitivity analysis, using single mean imputation in order to replace missing values on respective item scales. The results were comparable to those on the primary analysis set, the rotated component matrix is provided in the appendix (see Additional file 1).

**Table 4:** **Rotated factor loadings of SDSCA-G items 1–10 (related factors are printed in bold)**

**Item**	**1**	**2**	**3**	**4**
1	0.124	**0.896**	0.070	0.105
2	0.094	**0.913**	0.017	0.073
3	−0.149	**0.408**	0.380	−0.005
4	−0.106	0.223	0.313	**−0.644**
5	−0.229	0.215	0.133	**0.674**
6	−0.034	0.134	0.193	**0.680**
7	**0.936**	0.070	0.114	−0.074
8	**0.949**	0.063	0.110	−0.065
9	0.251	0.114	**0.720**	−0.006
10	0.061	−0.013	**0.837**	0.132
**Explained variance**				
By factor (%)	19.546	19.395	15.458	13.745
Cumulative (%)	19.546	38.942	54.399	68.144

### Confirmatory factor analysis

**Figure 1:**
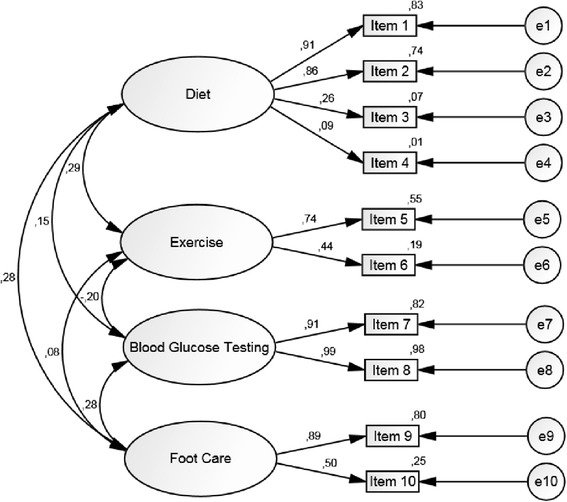
**CFA model of the 10 item questionnaire including latent variable correlations, standardized parameter estimates and squared multiple correlations.**

The χ^2^ of the model was 47.997, degrees of freedom were 29, and p = 0.015 based on the sample size of N = 237. The comparative fit index (CFI) of the model was 0.975 while the Tucker Lewis index (TLI) was 0.961. The root mean square error of approximation (RMSEA) was 0.053 and a standardized root mean square residual (SRMR) of 0.0526 was observed, yielding an acceptable model fit based on the cut-off values for CFI/TLI, RMSEA and SRMR recommended in [28]. The assessed model is displayed in Figure 1, along with the latent variable correlations, standardized parameter estimates and squared multiple correlations.

**Figure 2:**
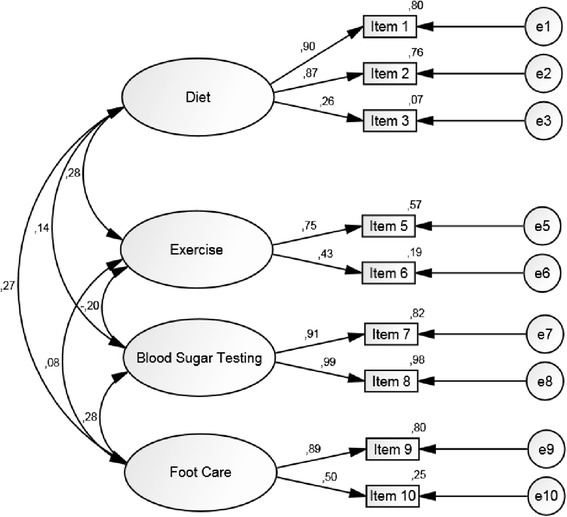
**CFA model of the questionnaire without item 4 including latent variable correlations, standardized parameter estimates and squared multiple correlations.**

One can observe here that item 4 has an astonishingly low factor loading of 0.09, thus making its contribution to the model fit somewhat questionable. Hence, we conducted a further CFA where Item 4 was excluded for the same analysis population of N = 237 (see Figure 2). The χ^2^ of this altered model was 29.895, degrees of freedom were 21, and p = 0.095. The model fit indices of this model were CFI = 0.988, TLI = 0.980, RMSEA = 0.042, SRMR = 0.0448. Hence, the model was improved in terms of both the model χ^2^ and the relevant model fit indices.

Just as for the exploratory factory analysis, the primary analysis was a complete case analysis of those N = 237 patients who had no missing values on the first 10 items of SDSCA-G. Hence, as a sensitivity analysis, both a CFA of the 10-item model and a CFA of the model without item 4 on the full set of N = 315 patients was conducted using single mean imputation. Again, these results deviated only slightly from the results on the primary analysis set. The CFA models and relevant model fit indices are provided in the appendix, respectively (see Additional files 2 and 3).

### Convergent construct validity

Convergent construct validity was assessed by calculating the correlation between the sum score of the first four items of the SDQ score and the SDSCA-G sum score. The outcome on these two scales was solely available for N = 308 patients in our sample, as the SDQ score could not be computed for 4 patients due to missing values on several items. Spearman’s ρ was chosen as correlation coefficient since the SDQ score was not assumed to be continuous. The analysis yielded an acceptable correlation of ρ = 0.644.

### Assessment of the influence of migration status and further predictor variables on the SDSCA-G sum score

The mean SDSCA-G sum score in migrants was 3.12 ± 1.12, while it was 3.23 ± 1.23 for non-migrants. Based on the result of a two-sample t-test, the difference between the two groups was not significant (p = 0.544), the corresponding 95% confidence interval was [−0.28, 0.51].

The multivariable linear model determined to find predictors for the SDSCA-G sum score had a R^2^ of 0.043 and was based on a sample size of N = 253. A positive influence was observed for women (β = 0.302, 95%-CI = [−0.001,0.606]), and the enrolment in a DMP (β = 0.272, 95%-CI = [−0.037,0.581]), while the influence of having migration status was slightly negative (β = −0.330, 95%-CI = [−0.764,0.103]). The impact of age (β = −0.007, 95%-CI = [−0.023,0.010]) and comorbidities (β =0.011, 95%-CI = [−0.083,0.105]) was close to negligible. However, none of the parameter estimates was significantly different from zero.

## Discussion

Overall, the German version of the SDSCA (SDSCA-G) demonstrated acceptable psychometric properties regarding reliability and validity for use in Germany, which were comparable to the original version [8]. Major challenges in translating and adapting the English SDSCA into German were not encountered and content validity could be assured. In addition, calculation of the convergent content validity showed an acceptable correlation between the SDQ and the SDSCA-G sum score. These findings suggest that the SDSCA-G is suitable for assessing self-management in patients with diabetes mellitus type 2 in Germany.

Toobert and colleagues [8] showed in their analysis that the predictors age and social desirability had a moderate influence on the original SDSCA sub scores. In contrast to these findings, our results revealed a slightly positive influence of being female and enrolled in a DMP. Moreover the influence of migration status was slightly negative on the SDSCA-G sum score, whereas the influence of age and comorbidity was close to negligible. However, none of these results were statistical significant and interfering the SDSCA-G sum score. Therefore, our findings support previous results from Toobert and colleagues [8] that the SDSCA can be generalized to different diabetes subpopulations including gender, DMP enrollment, migration status, age and number of comorbities.

Even though our results showed that the SDSCA-G performed well, it is important to note that item 4 caused some difficulties regarding the analysis of item characteristics as well as the evaluation of the scale’s structure and the model’s fit. The deletion of item 4 in the assessment of item characteristics and reliability yielded an increase in the SDSCA-G sum scale’s alpha coefficient. Similar results were found in other studies, which also evaluated the psychometric properties of a translated version of the SDSCA [29-32]. Even if our reliability coefficient of the SDSCA-G sum score was comparable to findings reported by other colleagues [7,19,29,32,33], it only just reached an acceptable value. As noted by Yin Xu and colleagues [33], the Cronbach’s alpha could be influenced by the number of items and the relationship among these items. In the SDSCA, diabetes self-management is measured by four different self-care activities (diet, exercise, blood glucose testing and foot care) and all of these activities have generally been found to be independent [8]. For example, a patient performing at a high level a healthy diet may not perform similarly on foot care. Therefore, the detection of a moderate Cronbach’s alpha value was not unexpected and appears to reflect the independency of each area of self-care activity. Furthermore, item 4 had a relatively low item-subscale and item-total correlation, whereas all other items showed acceptable item characteristics. This matches well with difficulties reported by Toobert and colleagues [8] in regard to internal consistency of the original SDSCA.

A further performed exploratory factor analysis (EFA) indicated a four factor construct of the translated questionnaire similar to its original English version (diet, exercise, blood glucose testing and foot care). Principal components with varimax rotation revealed a satisfactory percentage of total variance explained by the four factors. Although a four factor construct was suggested, our findings highlighted that item 4 formed a factor together with the two items assessing exercise (item 5 and 6), whereas all other items loaded on the intended factor. This result is in accordance with previous findings in the literature [31,32].

In addition, the confirmatory factor analysis (CFA) revealed an astonishingly low factor loading of item 4 which is in line with previous findings [29]. A further conducted CFA without item 4 showed improvements regarding the χ^2^ of the model as well as the relevant model fit indices. In general, the correlations between factors ranged from low to moderate, which appears to reflect again that diabetes self-management includes several independent aspects as well as supports previous results [29].

Overall, our findings underline the multidimensional construct of diabetes self-management with independent aspects of self-care activities. These results correlate fairly with previous findings [8,29]. Especially diet seems to have various components, which are not highly correlated. Nevertheless diet habits are a crucial factor in diabetes care [34,35]. Especially the intake of red meat seems to influence type 2 diabetes negatively [35,36]. Therefore, our results appear to reflect, that type 2 diabetes patients did not fully link their eating habits with their disease, especially in regard to high fat food. Toobert and colleagues [8], therefore, advise that specific eating habits may be assessed separately. Altogether, the present findings highlight the possible suggestion of omitting item 4 of the SDSCA-G to improve its psychometric properties as a reliable and valid tool assessing self-management of diabetes type 2 in Germany.

### Strengths and Limitations

The major strength of the study is the sample size (N = 315), which was adequate to achieve reliable statistical results of high external validity. Furthermore, the study sample showed a balanced gender ratio as well as included insulin-dependent and insulin-independent patients. For this reason, our results are especially reliable with regard to the influence of predictor variables on the SDSCA-G sum score.

However, the sample of this study consisted of type 2 diabetes patients with multiple co-occurring diseases and a higher mean age. Although, this is a diabetes population who would strongly benefit from a high level of self-management as well as needs to be considered when assessing the effectiveness of health care strategies for chronic conditions, nevertheless, there is a possibility that different results would have arisen if the focus had been on younger patients without additional chronic conditions and/or diabetes mellitus type 1.

A further limitation of the study as well as other studies assessing self-management is the lack of a “gold standard” comparison [37-39]. A reason for this might be that measuring self-management presents difficulties because of the many aspects that are inherent within this concept. Moreover, at the time when our study took part there was no other tool available in German, therefore we decided to use the SDQ as a comparison. This presented an acceptable internal consistency in a previous study with a larger sample (own unpublished data).

Furthermore, no retest was performed due to the cross-sectional study design. This led to a lack of information on the SDSCA-G’s stability or sensitivity to change.

## Conclusion

The findings of this study demonstrate that the revised German version of the SDSCA (SDSCA-G) with 10 items (see Additional file 4) is a reliable and valid tool assessing self-management in adults with type 2 diabetes independently of patient’s characteristics. A primary advantage is that the SDSCA-G is a relatively short and easy tool, which can be used in a study setting as well as in a busy clinical setting to collect appropriate data.

## Additional files

Additional file 1:
**Rotated factor loadings of SDSCA-G items 1–10 (related factors are printed in bold) under mean imputation.**
Additional file 2:
**CFA model of the 10 item questionnaire including latent variable correlations, standardized parameter estimates and squared multiple correlations under single mean imputation.** Degrees of freedom were 29, χ2 = 50.050 and p=,009. Respective fit measures were TLI = .963, CFI = .976, SRMR = 0.0507. Additional file 3:
**CFA model of the questionnaire without item 4 including latent variable correlations, standardized parameter estimates and squared multiple correlations under single mean imputation.** Degrees of freedom were 21, χ2 = 34.029 and p=,036. Respective fit measures were TLI = .974, CFI = .985, SRMR = 0.0463. Additional file 4:
**The revised German version of the Summary of Diabetes Self-care Activities Measure (SDSCA-G).**

## Abbreviations

CFA: Confirmatory factor analysis
CFI: Comparative fix index
DMP: Disease management program
EFA: Exploratory factor analysis
ICD: International classification of diseases
PCP: Primary care practice
RMSEA: Root mean square error of approximation
SDQ: Self-management Diabetes mellitus-Questionnaire
SDSCA: Summary of Diabetes Self-Care Activities measure
SDSCA-G: German version of the Summary of Diabetes Self-Care Activities measure
SRMR: Standardized root mean square residual
TLI: Tucker Lewis index
