# Supplementary material for: Assessing self-management in patients with diabetes mellitus type 2 in Germany: validation of a German version of the Summary of Diabetes Self-Care Activities measure (SDSCA-G)
Source: Health Qual Life Outcomes. 2014 Dec 18;12:185. doi: 10.1186/s12955-014-0185-1 (PMC4297436; doi:10.1186/s12955-014-0185-1)
Supplement: Additional file 1: — Rotated factor loadings of SDSCA-G items 1–10 (related factors are printed in bold) under mean imputation. [file 12955_2014_185_MOESM1_ESM.pdf]

**Additional file 1:** Rotated factor loadings of SDSCA-G items 1-10 (related factors are printed in bold) under mean imputation

| Item | 1 | 2 | 3 | 4 |
|---|---|---|---|---|
| 1 | **0.893** | 0.115 | 0.032 | 0.068 |
| 2 | **0.912** | 0.083 | -0.016 | 0.048 |
| 3 | **0.444** | -0.104 | 0.330 | 0.086 |
| 4 | 0.183 | -0.086 | 0.318 | **-0.560** |
| 5 | 0.235 | -0.210 | 0.114 | **0.692** |
| 6 | 0.104 | -0.001 | 0.170 | **0.718** |
| 7 | 0.059 | **0.949** | 0.089 | -0.066 |
| 8 | 0.054 | **0.938** | 0.101 | -0.033 |
| 9 | 0.112 | 0.200 | **0.747** | -0.061 |
| 10 | -0.016 | 0.035 | **0.825** | 0.141 |
| **Explained variance** | | | | |
| by factor (%) | 23.644 | 19.072 | 12.935 | 11.416 |
| cumulative (%) | 23.644 | 42.737 | 55.672 | 67.088 |
