# Supplementary material for: Assessing self-management in patients with diabetes mellitus type 2 in Germany: validation of a German version of the Summary of Diabetes Self-Care Activities measure (SDSCA-G)
Source: Health Qual Life Outcomes. 2014 Dec 18;12:185. doi: 10.1186/s12955-014-0185-1 (PMC4297436; doi:10.1186/s12955-014-0185-1)
Supplement: Additional file 2: — CFA model of the 10 item questionnaire including latent variable correlations, standardized parameter estimates and squared multiple correlations under single mean imputation. Degrees of freedom were 29, χ2 = 50.050 and p=,009. Respective fit measures were TLI = .963, CFI = .976, SRMR = 0.0507. [file 12955_2014_185_MOESM2_ESM.pdf]

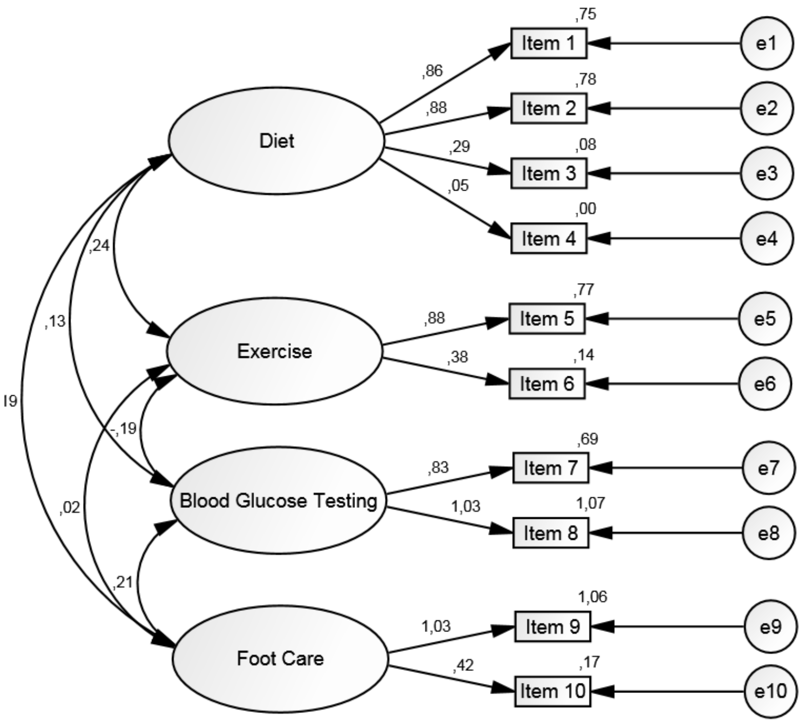

Additional file 2: CFA model of the 10 item questionnaire including latent variable correlations, standardized parameter estimates and squared multiple correlations under single mean imputation. Degrees of freedom were 29, χ^2=50.050 and p=,009. Respective fit measures were TLI=.963, CFI=.976, SRMR=0.0507.
