# Supplementary material for: Assessing self-management in patients with diabetes mellitus type 2 in Germany: validation of a German version of the Summary of Diabetes Self-Care Activities measure (SDSCA-G)
Source: Health Qual Life Outcomes. 2014 Dec 18;12:185. doi: 10.1186/s12955-014-0185-1 (PMC4297436; doi:10.1186/s12955-014-0185-1)
Supplement: Additional file 3: — CFA model of the questionnaire without item 4 including latent variable correlations, standardized parameter estimates and squared multiple correlations under single mean imputation. Degrees of freedom were 21, χ2 = 34.029 and p=,036. Respective fit measures were TLI = .974, CFI = .985, SRMR = 0.0463. [file 12955_2014_185_MOESM3_ESM.pdf]

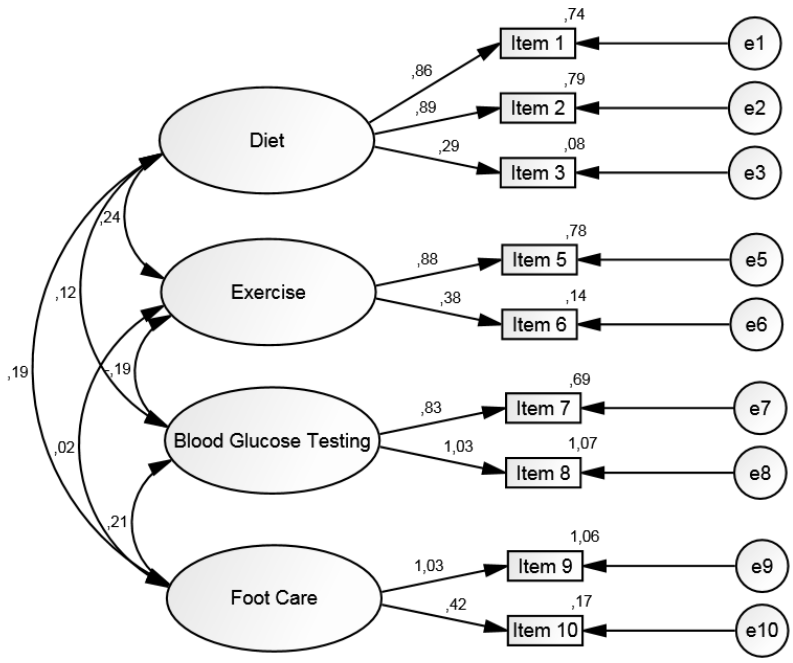

Additional file 3: CFA model of the questionnaire without item 4 including latent variable correlations, standardized parameter estimates and squared multiple correlations under single mean imputation. Degrees of freedom were 21, χ^2=34.029 and p=,036. Respective fit measures were TLI=.974, CFI=.985, SRMR=0.0463.
