# Supplementary material for: Assessing self-management in patients with diabetes mellitus type 2 in Germany: validation of a German version of the Summary of Diabetes Self-Care Activities measure (SDSCA-G)
Source: Health Qual Life Outcomes. 2014 Dec 18;12:185. doi: 10.1186/s12955-014-0185-1 (PMC4297436; doi:10.1186/s12955-014-0185-1)
Supplement: Additional file 4: — The revised German version of the Summary of Diabetes Self-care Activities Measure (SDSCA-G). [file 12955_2014_185_MOESM4_ESM.pdf]

Additional file 4: The revised German version of the Summary of Diabetes Self-care Activities Measure (SDSCA-G)

The Summary of Diabetes Self-care Activities Measure-German (SDSCA-G)

Die kommenden Fragen beziehen sich darauf, wie Sie in den vergangenen 7 Tagen mit Ihrer Zuckerkrankheit umgegangen sind. Falls Sie in den letzten 7 Tagen akut erkrankt waren, erinnern Sie sich bitte an die letzten 7 Tage, an denen Sie noch gesund waren.

Ernährung

An wie vielen von den letzten SIEBEN TAGEN haben Sie sich gesund ernährt?

0	1	2	3	4	5	6	7
☐	☐	☐	☐	☐	☐	☐	☐

An wie vielen TAGEN PRO WOCHE im letzten Monat haben Sie sich im Durchschnitt gesund ernährt?

0	1	2	3	4	5	6	7
☐	☐	☐	☐	☐	☐	☐	☐

An wie vielen der letzten SIEBEN TAGE haben Sie 5 oder mehr Portionen Obst oder Gemüse gegessen?

0	1	2	3	4	5	6	7
☐	☐	☐	☐	☐	☐	☐	☐

Körperliche Aktivität

An wie vielen der letzten SIEBEN TAGE haben Sie sich mindestens 30 Minuten lang am Stück körperlich betätigt (auch Spazierengehen, Garten- oder Hausarbeit)?

0	1	2	3	4	5	6	7
☐	☐	☐	☐	☐	☐	☐	☐

An wie vielen der letzten SIEBEN TAGE haben Sie Sport getrieben (z.B. Schwimmen, Nordic Walking, Radfahren)?

0	1	2	3	4	5	6	7
☐	☐	☐	☐	☐	☐	☐	☐

Blutzuckertest

An wie vielen der letzten SIEBEN TAGE haben Sie Ihren Blutzucker gemessen?

0	1	2	3	4	5	6	7
☐	☐	☐	☐	☐	☐	☐	☐

An wie vielen der letzten SIEBEN TAGE haben Sie Ihren Blutzucker so oft gemessen, wie man es Ihnen von medizinischer Seite empfohlen hat?

0	1	2	3	4	5	6	7
☐	☐	☐	☐	☐	☐	☐	☐

Fußpflege

An wie vielen der letzten SIEBEN TAGE haben Sie Ihre Füße untersucht?

0	1	2	3	4	5	6	7
☐	☐	☐	☐	☐	☐	☐	☐

An wie vielen der letzten SIEBEN TAGE haben Sie die Innenseite Ihrer Schuhe kontrolliert?

0	1	2	3	4	5	6	7
☐	☐	☐	☐	☐	☐	☐	☐

Rauchen

Haben Sie in den letzten SIEBEN TAGEN eine Zigarette – auch nur einen Zug – geraucht?

Ja	Nein
☐	☐

Wenn ja, wie viele Zigaretten haben Sie an einem normalen Tag durchschnittlich geraucht?

Anzahl der Zigaretten: | | |
